# Transurethrale Resektion von Blasentumoren (TUR-B)

**DOI:** 10.1007/s00120-021-01741-z

**Published:** 2022-01-04

**Authors:** D. Oswald, M. Pallauf, T. R. W. Herrmann, C. Netsch, B. Becker, K. Lehrich, A. Miernik, D. S. Schöb, K. D. Sievert, A. J. Gross, J. Westphal, L. Lusuardi, S. Deininger

**Affiliations:** 1 Universitätsklink für Urologie und Andrologie, Paracelsus Medizinische Universität Salzburg, Universitätsklinik für Urologie und Andrologie der PMU, Salzburger Landeskliniken, Müllner Hauptstraße 48, 5020 Salzburg, Österreich; 2 Urologie, Kantonsspital Frauenfeld, Frauenfeld, Schweiz; 3 Abteilung für Urologie, Asklepios Klinik Barmbek, Hamburg, Deutschland; 4 Klinik für Urologie, Vivantes Auguste-Viktoria-Klinikum, Berlin, Deutschland; 5 Medizinische Fakultät, Klinik für Urologie, Universitätsklinikum Freiburg, Freiburg, Deutschland; 6 UKOWL, Campus Klinikum Lippe, Detmold, Deutschland; 7 Klinik für Urologie, Kinderurologie und Urogynäkologie, Krankenhaus Maria Hilf der Alexianer GmbH, Krefeld, Deutschland

**Keywords:** Harnblasenkrebs, Nicht muskelinvasives Blasenkarzinom, Urothelkarzinom, Endourologie, Koagulation, Urinary bladder neoplasms, Non-muscle invasive bladder cancer, Urothelial cancer, Endourology, Coagulation

## Abstract

Die transurethrale Resektion von Blasengewebe (TUR-B) ist für die Diagnostik und Therapie bei Blasentumoren indiziert. Diese werden fragmentiert mittels diathermaler Schlinge abgetragen. Der Wundgrund wird zur Blutstillung koaguliert. Zu achten ist auf eine ausreichende Schnitttiefe, sodass die Detrusormuskulatur erfasst ist. Postoperativ kann zur Rezidivprophylaxe eine intravesikale Single-shot-Chemotherapie verabreicht werden. Methoden zur verbesserten Tumorvisualisation (insbesondere photodynamische Diagnostik) helfen, besonders bei multilokulärem Befund oder Carcinoma in situ (CIS) bessere Detektionsraten zu erreichen sowie das Rezidiv- und Progressionsrisiko zu senken. In Abhängigkeit von der Histologie ergibt sich das weitere Vorgehen: bei nicht muskelinvasivem Blasenkarzinom Nachsorge, adjuvante Instillationstherapie mittels Chemotherapie oder Bacillus Calmette-Guérin (BCG), die Nachresektion („second look TUR-B“), die Frühzystektomie oder bei muskelinvasivem Blasenkarzinom die Zystektomie oder (onkologisch nachrangig) die trimodale Therapie mit erneuter TUR‑B, Radiotherapie und Chemotherapie. Mögliche Komplikationen im Rahmen der TUR‑B sind v. a. Nachblutung mit Blasentamponade, extra- oder intraperitoneale Blasenperforation oder Infektionen des Urogenitaltrakts.

## Abstract (en)

Transurethral resection of bladder tumors (TURBT) is the standard of care for the diagnostics and primary treatment of bladder tumors. These are removed by fragmentation using loop diathermy. The resection area is coagulated for hemostasis. An important aspect is always a complete resection with an adequate amount of detrusor muscle in the specimen. Postoperative intravesical instillation of single-shot chemotherapy has been proven to reduce recurrence rates. Methods for improved tumor visualization (particularly photodynamic diagnostics) are used to enhance tumor detection rates particularly in multifocal tumors or carcinoma in situ (CIS). Thus, recurrence and progression rates can be reduced. Depending on the histological examination of the TURBT specimen, follow-up treatment for non-muscle invasive bladder tumors are adjuvant instillation treatment using chemotherapy or Bacillus Calmette-Guérin (BCG), second look TURBT and early cystectomy or for muscle invasive bladder tumors, radical cystectomy or (oncologically subordinate) trimodal treatment with renewed TURBT, radiotherapy and chemotherapy are indicated. Possible complications of TURBT include bleeding with bladder tamponade, extraperitoneal or intraperitoneal bladder perforation and infections of the urogenital tract.

## Lernziele

Nach Absolvieren dieser Fortbildungseinheit haben Sie Kenntnisse in folgenden Bereichen:

- Indikation – Diagnostik und Therapie: Sie wissen, in welchen Fällen Sie eine TUR‑B indizieren müssen.
- Operationstechnik: Sie kennen die Basics der TUR‑B sowie Tricks in speziellen Fällen, bei größeren Tumoren oder einer schwierigen Tumorlage.
- Erweiterte Tumorvisualisierung: Sie kennen die möglichen Techniken zur Optimierung der intraoperativen Tumordarstellung.
- Komplikationen und deren Management: Sie kennen die möglichen Komplikationen der TUR‑B und wissen, wie Sie diese beherrschen können.

## Einleitung

Das Urothelkarzinom der Harnblase ist die am elfthäufigsten gestellte Tumordiagnose weltweit mit altersstandardisierten Inzidenzraten (pro 100.000 im Jahr) von 9 bei Männern und 2,2 bei Frauen [1]. Der wichtigste Risikofaktor für das Urothelkarzinom der Harnblase ist der **Tabakkonsum**

Tabakkonsum,

der für etwa die Hälfte der Fälle verantwortlich ist [2].

Besteht der Verdacht auf einen Blasentumor, ist die transurethrale Resektion des Blasentumors (TUR-B) zur weiteren Diagnostik und Therapie indiziert [3].

## Indikationen der TUR-B – wichtige Aspekte in Diagnostik und Therapie

Die Indikationsstellung erfolgt meist durch eine **präoperative Zystoskopie**

Präoperative Zystoskopie

mit einer genauen Beschreibung von Tumoranzahl, -größe und -lage. Dies erleichtert die Operationsplanung mit Abschätzung der Operationszeit und der Wahl des Operateurs. Bei ausreichendem Verdacht kann die Operation aber auch auf Basis eines sonographischen oder computertomographischen Befunds erfolgen. Somit kann dem Patienten eine Zystoskopie ohne Narkose erspart werden. Auch eine positive Zytologie ohne zystoskopisches Korrelat kann bei unauffälliger Abklärung des oberen Harntrakts mittels 4‑Phasen-Kontrastmittelcomputertomographie (KM CT) eine TUR‑B notwendig machen.

### Diagnostische Aspekte

#### Pathologische Aufarbeitung

Gemäß dem histopathologischen Befund aus dem Resektionsmaterial der TUR‑B ergibt sich die individuelle Therapie- bzw. Nachsorgestrategie. Weitere Aspekte wie Multifokalität, Größe und Alter des Patienten müssen ebenso berücksichtigt werden [3].

In Europa sind über 90 % aller Blasentumoren **Urothelkarzinome**

Urothelkarzinome.

Diese können unterschiedliche Differenzierungsausprägungen aufweisen, die prognostisch unterschiedliche Bedeutung haben können [4]. Reine Plattenepithelkarzinome und Adenokarzinome (z. B. das Urachuskarzinom als Sonderform) sind deutlich seltener.

Zunächst wird gemäß dem TNM(Tumor/Nodus/Metastasen)-System 2017 [5] in **nicht muskelinvasiv** (pTa – exophytisch auf die Mukosa beschränkt, pT1 – bis in die Submukosa einwachsend, pTis [Carcinoma in situ, CIS] – schlecht differenziert intraepithelial wachsend) sowie **muskelinvasiv** (≥ pT2 –in die Detrusormuskulatur einwachsend) unterschieden.

Es existieren 2 unterschiedliche **Grading**-Systeme, die beide im histopathologischen Befund Erwähnung finden sollten. Das WHO(World Health Organization)-Grading 1973 teilt in G1, G2 und G3 ein, während das WHO-Grading 2004/2016 in PUNLMP („papillary urothelial neoplasm of low malignant potential“), Low-grade- und High-grade-Tumoren unterscheidet [6, 7].

Für die korrekte histologische Beurteilung muss **Detrusormuskulatur**

Detrusormuskulatur

im Präparat vorhanden sein. Fehlt diese, steigt das Risiko eines pathologischen Understagings und, darauf fußend, auch das einer schlechteren Prognose.

##### Cave

Das Vorhandensein von Detrusormuskulatur muss im histopathologischen Befund beschrieben sein. Es ist ein Marker für die Qualität der TUR-B.

Das Vorhandensein einer **lymphovaskulären Invasion**

Lymphovaskuläre Invasion

(LVI) sollte ebenso im histopathologischen Befund Erwähnung finden. Diese korreliert mit einer verschlechterten Prognose.

#### Risikostratifizierung

Das Urothelkarzinom der Harnblase ist in seiner Präsentation ausgesprochen heterogen mit großen Risikounterschieden im Hinblick auf Rezidiv- und Progressionsraten [8, 9].

Es wird in „low risk“, „intermediate risk“, „high risk“ und „highest risk“ unterteilt. In Abhängigkeit von diesen Gruppen kann die Nachsorge (für erstere), eine adjuvante intravesikale Instillationstherapie mit **Bacillus Calmette-Guérin**

Bacillus Calmette-Guérin

(BCG) bzw. Chemotherapie über 1 bis 3 Jahre oder eine Frühzystektomie empfohlen werden [3].

Diese Gruppen basieren hauptsächlich auf oben behandelten Kriterien des histopathologischen Befunds und stehen in direkter Abhängigkeit zur Qualität der durchgeführten TUR‑B.

Liegt ein muskelinvasives Blasenkarzinom vor, ist eine ausschließlich endoskopische Therapie im Regelfall nicht möglich. Therapie der Wahl ist dann die radikale Zystektomie mit Harnableitung. Als Alternative bei Ablehnung der Operation oder fehlender Operabilität aufgrund von Komorbiditäten kann eine **trimodale Therapie**

Trimodale Therapie

(TMT), bestehend aus TUR‑B, Chemotherapie und Radiotherapie, angeboten werden [10].

### Therapeutische Aspekte

#### Second-look-TUR-B

In ausgewählten Fällen ist eine zweite TUR‑B im kurzfristigen Abstand zur vorangegangenen TUR‑B indiziert. Diese wird allgemein als Second-look-TUR‑B bezeichnet. Ziel dieser Operation ist, eine möglichst vollständige Tumorresektion zu erreichen und ein **Understaging**

Understaging

durch die Erstresektion zu vermeiden [3].

Ein Second-look-Eingriff muss immer erfolgen, wenn der Operateur im Rahmen der Erstoperation die Einschätzung trifft, dass der sichtbare Tumor im Rahmen einer singulären Operation nicht vollständig resektabel ist. Ein nicht muskelinvasives Blasenkarzinom kann so auch in mehreren Sitzungen reseziert werden.

Eine weitere Indikation zur Nachresektion ist die fehlende Erfassung der Detrusormuskulatur im Präparat, wobei pTa-Low-grade/G1-Tumoren hier ausgenommen werden.

Bei pT1-Blasentumoren sollte immer eine Second-look-Resektion erfolgen, da das Risiko eines **Upstagings**

Upstagings

zu pT2 mit bis zu 30 % und das eines persistierenden Tumors mit über 50 % im Rahmen einer Zweitresektion in Studien bestätigt wurde [11]. Im Falle der pT1-Tumoren zeigte sich dies unabhängig vom Vorhandensein von Detrusormuskel im Präparat [12].

Im Falle eines pTa-High-grade-Tumors muss gemäß EAU(European Association of Urology)-Leitlinie nicht zwingend eine Second-look-TUR‑B erfolgen, sofern Detrusormuskel im Präparat nachgewiesen wurde [3]. Hierin unterscheidet sich die S3-Leitlinie und empfiehlt Nachresektionen bei allen High-grade-Tumoren [13].

##### Merke

Die 2nd look TUR‑B dient dazu ein Understaging durch die Erstresektion zu vermeiden.

Der Zeitpunkt der **Nachresektion**

Nachresektion

wird innerhalb von 2 bis 6 Wochen nach der Erstresektion empfohlen. Hierfür sprechen Daten, die bei Nachresektionen in diesem Zeitraum ein besseres rezidivfreies bzw. progressionsfreies Überleben belegen [14].

Die Second-look-TUR‑B kann Rückschluss auf die Qualität der Erstresektion erlauben.

#### Palliative TUR-B

Im Falle des muskelinvasiven Blasenkarzinoms ist die radikale Zystektomie oder nachrangig die trimodale Therapie (TMT) indiziert. Werden diese abgelehnt oder ist der Patient hierfür aufgrund von Komorbiditäten oder Allgemeinzustand nicht geeignet, kann in Ausnahmefällen von der rezidivierenden „palliativen“ TUR‑B Gebrauch gemacht werden. Primäre Ziele sind hierbei die möglichst nachhaltige **Symptomkontrolle**

Symptomkontrolle

sowie die Tumorresektion „ins Niveau“, d. h. auf Höhe des Blasenschleimhautniveaus. Selbst bei diesen Patienten wird nicht selten die Durchführung einer Zystektomie aufgrund von lokalen Beschwerden oder unstillbaren, rezidivierenden Blutungen notwendig [15].

#### TUR-B als Teil der trimodalen Therapie des muskelinvasiven Blasenkarzinoms

Die effektive Radiotherapie der Harnblase bedingt die maximale Reduktion der Tumorlast, weshalb die TUR‑B mit Tumorresektion ins Blasenschleimhautniveau oder tiefer vor Beginn der kombinierten Radiochemotherapie notwendig ist.

## Technik der TUR-B

Für eine Übersicht über die Technik der TUR‑B sei auf die einschlägige Literatur verwiesen [16, 17, 18].

### Lagerung

Der Patient liegt in **Steinschnittlage**

Steinschnittlage.

Das Verfahren kann in Allgemeinnarkose oder in Spinalanästhesie durchgeführt werden. In Ausnahmefällen kann eine Resektion auch in Lokalanästhesie durchgeführt werden.

### Intraoperative Zystoskopie und klinische Untersuchung

Beim Mann kann die digital-rektale, bei der Frau die bimanuelle Untersuchung Hinweise auf ein lokal fortgeschrittenes Karzinom liefern.

Intraoperativ erfolgt zunächst eine zystoskopische Untersuchung des Patienten. Beim Mann wird hierfür zunächst eine 0°-Optik zur Beurteilung der Urethra, des Schließmuskels und der prostatischen Harnröhre verwendet. Für die eigentliche Zystoskopie wird im Regelfall eine 70°-Optik verwendet. Dies kann jedoch nach Präferenz des Operateurs variieren.

Im Operationsbericht müssen Tumoranzahl und -größe sowie die genaue Lokalisation beschrieben sein. Dies ist zusätzlich zum histopathologischen Befund wichtig für das **Risikoassessment**

Risikoassessment

hinsichtlich Rezidiv- und Progressionsrisiko [9, 19]. Zur Abschätzung der Größe kann die Schlingenbreite benutzt werden, die etwa zwischen 2,5 und 10 mm variieren kann.

Die Beurteilung der Ostien (Lage, Konfiguration, Ejakulation von [klarem] Harn) kann Hinweise auf eine Pathologie des oberen Harntrakts liefern. Eine retrograde Darstellung oder Harnleiterschienung sollte bei Tumornachweis in der Blase nur mit Zurückhaltung erfolgen. Im Falle einer begleitenden Harnstauungsniere ist die **Nephrostomieanlage**

Nephrostomieanlage

mit antegrader Abklärung vorzuziehen, wobei die Datenlage hierzu schwach ist [20].

Bei unklaren Rötungen bzw. Verdacht auf CIS kann eine Zytologie abgenommen werden, falls nicht präoperativ im Rahmen der Primärdiagnostik geschehen. Mittels **Ureterkatheter**

Ureterkatheter

kann auch eine seitendifferenzierte Zytologie des oberen Harntrakts gewonnen werden.

### Resektionstechnik

Vor Resektionsbeginn ist es wichtig, eine Strategie für die gesamte Resektion anhand der zystoskopisch erhobenen Befunde zu haben. Insbesondere im Falle einer durch Blutung eingeschränkten Sicht kann so ein systematisches und sicheres Fortführen der Operation gewährleistet werden.

Die Resektion wird mit einer monopolaren oder bipolaren diathermalen Resektionsschlinge durchgeführt. Diese ist im Regelfall 90° zum Instrumentschaft gebogen, für die Hinterwand gibt es zudem Schlingen mit 45°-Winkel. Während der Resektion ist auf eine ausreichende Blasenfüllung zu achten. Um eine Überfüllung mit konsekutiver Ausdünnung der Blasenwand zu vermeiden, empfiehlt es sich, den Spülstrom bei halber Stärke laufen zu lassen. So kann das Risiko einer Blasenperforation vermindert werden. Hilfreich sind auch **Rückspülresektoskope**

Rückspülresektoskope,

die bei konkomitantem Zu- und Ablauf eine kontinuierliche Blasenfüllung erlauben.

Die Resektionsschlinge sollte vor Berührung mit dem Gewebe bereits elektrisch aktiviert werden. Dies ist insbesondere bei bipolaren Systemen wichtig. Die Aktivierung ermöglicht eine gute Kontrolle der Eindringtiefe trotz im Allgemeinen fehlender haptischer Kontrolle während der Interaktion mit dem Gewebe.

#### Merke

Die Resektionsschlinge sollte vor Berührung mit dem Gewebe bereits aktiviert werden.

Im Allgemeinen erfolgt die Resektion retrograd, also in Richtung des Operateurs. Eine antegrade Resektion ist jedoch ebenso möglich und kommt beispielsweise im Rahmen der En-bloc-Resektion zur Anwendung.

In Abhängigkeit von Tumorlage und Wölbung der Blasenwand können unterschiedliche Resektionstechniken zum Einsatz kommen. Die Resektion erfolgt durch das Bewegen der Resektionsschlinge. Durch ein konkomitantes Absenken und Wiederanheben des Instruments kann eine gute Kontrolle über eine gleichförmige Resektionstiefe bei gewölbter Blasenwand erfolgen. Insbesondere bei Tumoren der Hinterwand kann es hilfreich sein, die Schlinge zu fixieren, um die Resektion vollständig durch Bewegen des Instrumentenschafts durchzuführen.

Ein Trick hierbei ist, auch unterschiedlich gebogene Schlingen zu verwenden bzw. diese selbst manuell zu verformen. Bei Tumoren der Vorderwand und des Blasendachs kann mittels Drucks auf die Bauchdecke mit der zweiten, nichtdominanten Hand oder alternativ durch Assistenz eine bessere Resektionsposition erreicht werden.

Primäres Ziel ist die vollständige Abtragung von exophytischen Tumorformationen bis in die Detrusorschicht. Hierbei können 3 strategisch unterschiedliche Varianten unterschieden werden, und zwar die horizontale, die vertikale sowie die Resektion am Tumorstiel.

Die schrittweise horizontale Abtragung ist für kleinere Tumoren vorgesehen. Da das Tumorbett mit der Gefäßzufuhr erst gegen Ende der Resektion erreicht wird, ist im Falle einer Blutung die Hämostase erschwert durchführbar.

Größere bzw. solide Tumoren sollten mit vertikalen Schnitten, also vom Rand beginnend, reseziert werden. Hierbei wird Schritt für Schritt der Tumorgrund freigelegt und hämostatisch versorgt, sodass trotz großer Tumormassen eine suffiziente Kontrolle bei Blutungen möglich ist.

Gestielte Tumore können an der Basis „en bloc“ abgetrennt werden. Je nach Tumorgröße kann jedoch eine weitere Fragmentierung zur Evakuation des Präparats notwendig sein.

#### Biopsien, kleine Tumorbefunde

Liegt der Verdacht auf ein CIS vor (z. B. flächige diffuse Rötungen, positive Spülzytologie) sollte eine **4‑Quadranten-Biopsie**

4‑Quadranten-Biopsie

oder eine **PDD(photodynamische Diagnostik)-geführte Biopsie**

PDD-geführte Biopsie

durchgeführt werden. Hierfür kann die Biopsiezange anstatt einer Resektionsschlinge verwendet werden, wodurch Koagulationsartefakte vermieden werden.

Eine Biopsie der prostatischen Harnröhre kann bei vorangegangenem Befall dieser Lokalisation oder (ausgeprägtem) Befall am Blasenhals notwendig sein. Ebenso hilfreich kann diese zur weiteren Operationsplanung bei absehbar notwendiger Zystektomie sein.

Bei ausgedehntem Tumorbefall, der bei vollständiger Resektion eine ausgeprägte Vernarbung der Blasenwand zur Folge hätte, kann auf eine **Elektrokoagulation/-vaporisation**

Elektrokoagulation/-vaporisation

zurückgegriffen werden. D. h., Tumorausläufer und -rasen werden isoliert und ohne Gewebsresektion elektrothermisch behandelt, um die darunterliegenden Schichten der Harnblase zu schonen. Insbesondere bei bekanntem rezidivierenden pTa-Low-grade-Tumor stellt diese Therapieform eine Option dar – wobei allerdings keine Histologie gewonnen wird.

Die einzelnen resezierten Tumoren sollten getrennt histopathologisch untersucht werden. Zudem empfiehlt es sich, den exophytischen Tumoranteil und den Tumorgrund (Fragestellung: Detrusor?) getrennt einzusenden.

### Tumorlokalisationen

Bei Resektionen an der Blasenseitenwand muss an den **Obturatoriusreflex**

Obturatoriusreflex

gedacht werden, der durch eine spontane Adduktorenkontraktion der Beine zu einer iatrogenen Blasenperforation durch die Schlinge führen kann. Bei bekannter Tumorlokalisation an der Seitenwand sollte prä- oder intraoperativ mit der Anästhesie Rücksprache gehalten werden. Die Relaxierung des Patienten oder ein selektiver Obturatoriusblock sind Mittel, um einen Obturatoriusreflex zu unterdrücken. Bei der Resektion ist auf kleine, schnelle Schnitte und eine geringere Blasenfüllung zu achten. Bipolarer Strom senkt im Verhältnis zum monopolaren Strom das Risiko, den Reflex auszulösen.

#### Cave

Bei Resektion im Bereich der Blasenseitenwand kann es zum Obturatoriusreflex kommen, der zur Blasenperforation führen kann.

Im Bereich der Blasenhinter- sowie -vorderwand ist besondere Vorsicht geboten und auf eine geringere Blasenfüllung zu achten, da ein erhöhtes Risiko für eine intraperitoneale Perforation besteht (das Komplikationsmanagement wird im Verlauf genauer diskutiert).

Bei ostialen Tumoren kann es notwendig sein, das Ostium zu resezieren. Kranial davon sollte eine vorsichtige Resektion erfolgen, um den Harnleiter zu schonen. Eine vollständige Tumorresektion sowie das Vorhandensein von Detrusormuskel im Präparat haben jedoch Priorität. Bei Wachstum in den Harnleiter kann auch eine ggf. zweizeitige **Ureterorenoskopie**

Ureterorenoskopie

mit Tumorlaserung und/oder Biopsieentnahme notwendig sein. Durch das Vermeiden von übermäßiger Koagulation um das Ostium können narbige Strikturen und daraus resultierende Harnabflussstörungen vermieden werden. Je nach intraoperativem Befund kann eine direkte Harnableitung oder aber eine postoperative sonographische Kontrolle notwendig sein.

Im Bereich des Blasenhalses des Mannes sowie der prostatischen Harnröhre muss meist Prostatagewebe mitreseziert werden. Insbesondere basal ist aufgrund der ausgeprägten Gefäßversorgung auf eine genaue Blutstillung zu achten. Je nach Ausmaß der Resektion muss mit Komplikationen wie der **retrograden Ejakulation**

Retrograde Ejakulation

gerechnet werden.

Eine Resektion in einem Blasendivertikel ist nicht immer vollständig und sicher möglich, da dieses meistens nur aus Mukosa und Submukosa besteht und damit die Perforationsgefahr durch die Resektion deutlich erhöht ist. In Abhängigkeit vom histopathologischen Befund kann eine Blasenteilresektion bis hin zur radikalen Zystektomie notwendig sein.

### Hämostase

Die effektivste Blutstillung erfolgt auf Tumorbasisniveau im gesunden Gewebe. Eine Koagulation im Tumorgewebe ist weniger effektiv. Spritzende arterielle Blutungen, die als Blutfahne sichtbar werden, sollten jedoch immer sofort punktuell koaguliert werden. Dies hilft insbesondere bei größeren Tumoren, eine gute Sicht intraoperativ zu bewahren. Diffuse venöse Blutungen können nach Ende der Resektion bzw. Evakuation systematisch koaguliert werden. Hier empfiehlt sich die Koagulation des gesamten Wundgrunds inklusive der Schnittränder, da hier die Gefäße zwischen Schleimhaut und Muskelschicht freiliegen und postoperativ ohne intravesikalen Druck zu bluten beginnen können. Vor Beendigung des Eingriffs sollte bei geringer Blasenfüllung ohne Spülstrom die Hämostase zystoskopisch verifiziert werden.

Postoperativ ist die Einlage eines **Dauerkatheters**

Dauerkatheter

für 1 bis 3 Tage, je nach Resektionstiefe, notwendig. Hierdurch erfolgen eine Schnittrandadaption und eine beschleunigte Wundheilung. Bei nicht vollständig klarem Harn sollte eine Rundspülung via **Spülkatheter**

Spülkatheter

durchgeführt werden, um Koagelbildung zu vermeiden. Da die postoperative Blasenirrigation mittels Natriumchloridlösung nachgewiesenermaßen das postoperative Rezidivrisiko in ähnlichem Ausmaß wie bei der postoperativen Chemotherapieinstillation senkt, empfiehlt sich die routinemäßige Verwendung von Spülkathetern und postoperativer Blasenirrigation zumindest für einige Stunden [10].

### Postoperative intravesikale Single-shot-Chemotherapie

Nach TUR‑B kann im Falle von Primärtumoren, Intermediate-risk-Tumoren mit weniger als 1 Rezidiv pro vorangegangenem Jahr bzw. einem EORTC(European Organisation for Research and Treatment of Cancer)-Risikoscore unter 5 eine einmalige intravesikale Chemotherapie verabreicht werden. Dies senkt das Rezidivrisiko um 14 % durch das Eliminieren von zirkulierenden Tumorzellen. Die Verabreichung sollte innerhalb von 24 h mit einer Einwirkdauer von 1 bis 2 h erfolgen. In dieser Zeit ist die Blasenirrigation selbstverständlich zu pausieren. Mögliche Therapeutika sind **Mitomycin C**

Mitomycin C,

**Epirubicin**

Epirubicin

und **Doxyrubicin**

Doxyrubicin.

Kontraindikationen sind eine suspizierte Blasenperforation sowie eine spülpflichtige Makrohämaturie [3].

### En-bloc-Resektion von Blasentumoren

Die konventionelle TUR‑B mit fragmentierter Tumorabtragung erscheint unter vielen Aspekten als suboptimal für die Behandlung des primären nicht muskelinvasiven Blasenkarzinoms. Es wurde postuliert, dass die Fragmentierung die Streuung von Tumorzellen und in der Folge deren Einnistung in gesundem Urothel an anderer Stelle fördert. Somit könnte die fraktionierte Resektion die hohen Rezidivraten mitbegründen. Zudem behindern die Fragmentierung und die Koagulation eine exakte pathologische Beurteilung von Invasionstiefe und freien Resektionsrändern sowie die räumliche Zuordnung des Präparats.

Um diese Probleme zu lösen, wurde die En-bloc-Resektion von Blasentumoren (ERBT) entwickelt und in verschiedenen Studien untersucht. Erste Konzepte wurden bereits 1997 mit hochfrequentem Strom und 1998 mit Laser als Energiequelle beschrieben.

Die Schlüsselkonzepte der ERBT umfassen die verlässliche und ausreichende Resektion von Detrusormuskel sowie beurteilbare Resektionsränder der Resektionspräparate.

Die ERBT wurde in verschiedenen Techniken beschrieben. Im Allgemeinen kann sie mit monopolarem oder bipolarem Strom oder mit verschiedenen **Lasern**

Laser

(Holmium, Thulium etc.) durchgeführt werden. Für Erstere wurden Techniken mit verschiedenen Schlingen beschrieben, wobei die meistverwendete eine rechteckige Schlinge ist. Auch die konventionelle TUR-B-Schlinge oder eine Dornsonde wurden angewandt. Eine eigenständige Technik orientiert sich an der Abtragung von Darmpolypen im Rahmen der Koloskopie. Hierbei wird der Tumor auf Höhe der Submukosa unterspritzt und somit über das Schleimhautniveau abgehoben, bevor er mit Strom „en bloc“ abgetrennt wird.

Im Regelfall ist die Resektionsrichtung der ERBT antegrad, also vom Operateur auf den Tumor zu.

Ein Problem der ERBT ist die intakte Bergung des „en bloc“ resezierten Präparats aus der Blase, das insbesondere größere Tumoren betrifft. Die En-bloc-Resektion von größeren Tumoren ist technisch grundsätzlich möglich. Für die Evakuation wurden unterschiedliche Techniken wie das Fassen des Präparats mit der Resektionsschlinge oder die Verwendung von **Endobags**

Endobags,

ähnlich den aus der laparoskopischen Chirurgie bekannten, beschrieben [21, 22, 23].

Die aktuelle Leitline der EAU empfiehlt die konventionelle TUR‑B sowie die ERBT gleichermaßen bei Verdacht auf Blasentumor [3]. Es fehlen jedoch noch prospektive, randomisierte Daten zum Vergleich mit der konventionellen Methode.

## Erweiterte Techniken der Tumorvisualisierung

Die routinemäßige Zystoskopie bzw. TUR‑B wird mit Weißlicht (WL) durchgeführt. Es stehen jedoch verschiedene Techniken zur verbesserten Visualisierung von Blasentumoren zur Verfügung. Dies ist insbesondere im Falle multilokulärer Tumoren oder bei Verdacht auf CIS von Relevanz und kann zu deutlich erhöhten Detektionsraten führen. Verschiedene etablierte Techniken stehen zur Verfügung.

### Photodynamische Diagnostik

Eine Stunde präoperativ wird **5‑Aminolävulinsäure**

5‑Aminolävulinsäure

(ALA) oder **Hexaminolävulinsäure**

Hexaminolävulinsäure

(HAL) via Einmalkatheterismus intravesikal instilliert. Diese Aminosäuren stellen Bestandteile der Hämbiosynthese dar. Im Tumorgewebe wird das photosensitive Molekül langsamer weiter verstoffwechselt, sodass es dort temporär akkumuliert. Unter Blaulichtvisualisation mittels separater Lichtquelle entsteht ein Fluoreszenzeffekt, durch den Blasentumoren sich rosa von der violett dargestellten regulären Blasenmukosa abheben.

Mit PDD können insbesondere Tumorausläufer, kleine multilokuläre Tumoren oder ein flächiges CIS besser erkannt werden. Sensitivitätsvorteile von 92 % gegenüber 71 % in WL konnten so auf Biopsieebene gezeigt werden [24]. Dies geht auf Kosten einer höheren falsch-positiven Rate auf Visualisationsebene, da entzündliche Veränderungen, vorangegangene BCG-Instillationen (innerhalb von 3 Monaten) sowie TUR‑B den Fluoreszenzeffekt beeinflussen können [25, 26].

Weitere Studien konnten zudem zeigen, dass die Verwendung von PDD das Rezidivrisiko senkt [27, 28].

#### Merke

PDD ist insbesondere bei multilokulärem Tumorbefall oder Verdacht auf CIS hilfreich zur besseren Unterscheidung von gesundem und tumorösem Gewebe.

### Narrow Band Imaging

Narrow Band Imaging (NBI) verschärft den Kontrast zwischen hypervaskularisiertem Gewebe (im Regelfall Tumor) zur normalen Blasenschleimhaut. Eine verbesserte Detektion im Vergleich zur WL-Zystoskopie konnte sowohl für die flexible Zystoskopie als auch für die TUR‑B gezeigt werden [29, 30, 31, 32]. Eine verringerte Rezidivrate bestätigte sich nur für die Subgruppe der Low-risk-Tumoren nach 3 und 12 Monaten [33].

### Weitere Methoden

Eine weitere Methode nutzt 4 verschiedene Lichtspektren zur unterschiedlichen Kontrastierung von Tumorgewebe und -vaskularisation [34]. Zur Validierung dieser Technik fehlen prospektive Daten.

## Komplikationen und deren Management

Für eine Übersicht über Komplikationen und deren Management sei auf die einschlägige Literatur verwiesen [16].

### Nachblutung und Blasentamponade

Eine Nachblutung kann durch eine ausführliche Koagulation des Wundgrunds sowie durch das Ausspülen aller Koagel vermieden werden.

Das initiale Management beruht auf der Einlage eines dreilumigen Spülkatheters und anschließender Koagelevakuation und Dauerspülung. Somit wird das Entstehen einer neuerlichen Blasentamponade verhindert.

Im Falle blasenhalsnaher Resektionen bzw. Nachblutungen können Maßnahmen wie im Rahmen der TUR-Prostata (TUR-P) versucht werden: Aufblocken des Ballons über 10 ml, Zug (z. B. 150 ml) bzw. eine Schleife als Kompresse um den Katheter sind denkbare Optionen.

Bei anhaltenden Blutungen muss frühzeitig in Narkose revidiert werden. Auch hier ist eine gründliche Evakuation der Koagel bzw. eine Blasentamponade essenziell. Anschließend kann der Wundgrund erneut koaguliert bzw. nachreseziert werden.

Arterielle Blutungen müssen bei schnell wechselnder, pulsierender Harnfarbe im Spülschlauch mit hellroten Wolken suspiziert werden. Ein konservatives Management ist dann meist nicht zielführend.

### Blasenperforation – intraperitoneal und extraperitoneal

Risikofaktoren sind Alter sowie weibliches Geschlecht (verhältnismäßig dünnere Blasenwand). Auslösende Ursachen können eine Hyperdistension der Blase durch großes Füllungsvolumen, eine Reizung des Nervus obturatorius oder eine instrumentelle Perforation durch eine tiefe Resektion sein.

Auffallen kann eine Perforation durch eine **inadäquate Blasenfüllung**

Inadäquate Blasenfüllung

bei vollem Spülstrom und sistierendem Abfluss, durch Größenzunahme des Abdomens oder visuell durch Sichtbarkeit von Fettgewebe.

Die Therapie der extraperitonealen Perforation ist die konsequente Harnableitung der Harnblase mittels Dauerkatheter über eine variable Anzahl von Tagen in Abhängigkeit vom Ausmaß der Perforation. So sind die Wundränder adaptiert und können sekundär abheilen. In jedem Fall ist auf eine gute Blutstillung auch in der Tiefe zu achten. Auf eine postoperative Irrigation sollte nach Möglichkeit verzichtet werden. Je nach Ausmaß der Perforation kann vor Katheterentfernung eine **Zystographie**

Zystographie

durchgeführt werden. Auch diagnostisch kann bei unklarer Lage der Perforation und Beschwerden ein Zystogramm wegweisend sein und zum Ausschluss einer intraperitonealen Perforation dienen. Im Falle der intraperitonealen Perforation muss eine offene Blasenübernähung via **Sectio alta**

Sectio alta

erfolgen.

#### Cave

Bei Resektion im Bereich des Blasendachs ist besondere Vorsicht geboten, da eine Perforation hier nach intraperitoneal führt.

Wurde Spülflüssigkeit intraabdominell eingeschwemmt, sollte eine regelmäßige laborchemische Überprüfung des Elektrolythaushalts erfolgen.

### TUR-Syndrom

Bei verlängerter Resektionszeit kann es bei monopolarer Resektion zum Einspülen von Spülflüssigkeit in den Blutkreislauf kommen. Dies führt zu einer **hypotonen Hyperhydratation**

Hypotone Hyperhydratation

(Hypervolämie, Hyponatriämie, Hyperkaliämie) mit möglichen schweren Folgeschäden wie Hirn- und Lungenödembildung, Hämolyse, Bradykardie und Nierenversagen. Bei Verwendung von bipolaren Systemen sowie isotoner Kochsalzlösung als Spülflüssigkeit kann das Risiko von schweren Nebenwirkungen deutlich verringert werden, eine Hyperhydratation ist in seltenen Fällen jedoch auch möglich.

### Ostienverletzung

Im Falle einer ungewollten Ostienverletzung kann eine Harnableitung mittels **Harnleiterschienung**

Harnleiterschienung

oder **Nephrostomie**

Nephrostomie

notwendig sein. Eine postoperative sonographische Kontrolle sowie eine laborchemische Kontrolle der Nierenfunktionsparameter sollten erfolgen.

Vernarbungen der Ostien können zur fixierten Obstruktion führen und eine dauerhafte Harnableitung (Nephrostomie/**Doppel-J-Katheter**

Doppel-J-Katheter)

oder eine operative Revision mittels Harnleiterneuimplantation bedingen.

### Infektionen

Postoperative Harnwegsinfektionen können durch präoperative Urinkontrolle, Urinkulturanlage sowie testgerechte Antibiose meist verhindert werden. Der postoperative Dauerkatheter garantiert die Harnableitung. Sollten trotzdem Infektzeichen auftreten, muss gemäß der lokalen Resistenzlage und den Leitlinien antibiotisch behandelt werden. Eine perioperative Prophylaxe ist grundsätzlich nur bei erhöhtem Risiko für eine postoperative Sepsis empfohlen [3].

### Narbenkomplikationen (Schrumpfblase)

Eine Schrumpfblase aufgrund von wiederholten, ausgedehnten Resektionen ist selten, kann in Ausnahmefällen bei ausgeprägter Beschwerdesymptomatik aber bis zur Zystektomie führen.

### Retrograde Ejakulation und BPS-Exazerbation (Resektion der prostatischen Harnröhre)

Bei Tumorresektionen in der prostatischen Harnröhre kann es analog zur TUR‑P zur retrograden Ejakulation kommen. Eine ausführliche präoperative Aufklärung ist geboten. Eine vorbestehende Symptomatik bei **benignem Prostatasyndrom**

Benignes Prostatasyndrom

(BPS) kann zudem insbesondere bei rezidivierenden transurethralen Eingriffen exazerbiert werden.

## Fazit für die Praxis

- Die transurethrale Resektion von Blasentumoren (TUR-B) ist der Goldstandard zur Diagnostik und Ersttherapie des Blasentumors. Die Indikation kann bei zystoskopischem, sonographischem oder computertomographischem Verdacht gestellt werden.
- Die TUR‑B kann zusätzlich im Rahmen einer Nachresektion bei unvollständiger Erstresektion oder Hochrisikotumor, im Rahmen einer trimodalen Therapie sowie palliativ zur Symptomkontrolle erfolgen.
- Eine gut durchgeführte TUR‑B beinhaltet die vollständige Resektion des Tumors bis in die Muskelschicht, sodass Detrusormuskulatur im Präparat enthalten ist.
- Vor Beginn der Resektion sollte ein Plan festgelegt werden, nachdem systematisch alle Befunde reseziert werden.
- Eine adäquate Blasenfüllung hilft, eine Perforation zu vermeiden.
- Eine ausreichende Hämostase mittels Koagulation sowie suffizienten Spülstroms sorgt für ausreichend Sicht während der Resektion insbesondere bei größeren Tumoren.
- Schwierige Lokalisationen von Blasentumoren können durch Variation in der Blasenfüllung, Druck auf das Abdomen oder unterschiedlich gebogene Schlingen erreicht werden.
- Photodynamische Diagnostik und Narrow Band Imaging sind erweitere Visualisierungsmöglichkeiten, die helfen, Tumoren und deren Ausbreitung besser zu erkennen. Besonders hilfreich sind diese bei multilokulären Tumoren oder Verdacht auf ein Carcinoma in situ.
- Die schwerwiegendste Komplikation ist die Blasenperforation. Falls diese extraperitoneal ist, kann sie meistens konservativ mit einer längeren Katheterliegezeit beherrscht werden. Eine intraperitoneale Perforation muss im Regelfall mittels Sectio alta und Blasenübernähung behandelt werden.
- Ist das Ostium durch den Blasentumor verlegt, muss es soweit reseziert werden, dass der Tumor vollständig entfernt ist. Im Falle einer Obstruktion kann eine Nephrostomie gestochen oder eine Harnleiterschiene gelegt werden.

## CME-Fragebogen

Ein Patient kommt zu Ihnen in die Spezialambulanz zur Besprechung des weiteren Vorgehens nach erfolgter transurethraler Resektion von Blasengewebe (TUR-B) vor 2 Wochen. Der histopathologische Befund ergab ein Urothelkarzinom der Harnblase (pTa „high grade“ G3). Detrusormuskulatur konnte *nicht *nachgewiesen werden.

Da das Stadium pTa vorliegt, besprechen Sie die weitere Nachsorge mit regelmäßigen Zystoskopien.

Bei High-grade-G3-Befund indizieren Sie die Zystektomie zur frühen Kontrolle bei hochgradig aggressivem Tumor.

Aufgrund der fehlenden Detrusormuskulatur indizieren Sie eine Second-look-TUR‑B, um einem Understaging vorzubeugen.

Da dies bei Urothelkarzinomen der Harnblase grundsätzlich indiziert ist, planen Sie eine Second-look-TUR‑B, um einem Understaging vorzubeugen.

Bei pTa-High-grade-Befund besprechen Sie nach Risikostratifizierung eine adjuvante Instillationstherapie gemäß den gängigen Leitlinien.

Ein Patient wird Ihnen vom Hausarzt mit auswärtigem Zufallsbefund eines 3 cm großen Blasentumors an der Harnblasenhinterwand in der Computertomographie in die urologische Ambulanz zugewiesen. Wie gehen Sie vor?

Sie empfehlen die transurethrale Resektion von Blasengewebe (TUR-B) nach Durchsicht der vorliegenden Bilder, da sie einen Blasentumor annehmen.

Sie verifizieren den Befund mittels Ultraschalls, erst dann können Sie die Indikation zur TUR‑B stellen.

Da kein ausreichender Verdacht besteht, führen Sie eine Zystoskopie durch, in der sich ein 3 cm papillärer Blasentumor bestätigt. Erst dann indizieren sie die TUR‑B.

Sie indizieren eine zystoskopische Verlaufskontrolle in 3 Monaten. Je nach Verlauf kann dann eine TUR‑B stattfinden.

Sie indizieren eine computertomographische Verlaufskontrolle in 3 Monaten. Je nach Verlauf kann dann eine TUR‑B stattfinden.

Sie beginnen eine transurethrale Resektion von Blasengewebe (TUR-B). Vorliegend ist der Befund des niedergelassenen Urologen, der multilokuläre Blasentumoren am Blasenboden und der Hinterwand beschreibt. Nach unauffälliger Urethroskopie schauen Sie in die Harnblase. Wie gehen Sie weiter vor?

Sie sehen unmittelbar den ersten Tumor und beginnen mit der Resektion, damit Sie ihn später nicht übersehen. Auch die weiteren Tumoren werden unmittelbar nach Detektion reseziert.

Sie führen eine Übersichtszystoskopie durch, merken sich die Tumorlokalisationen und resezieren diese dann der Reihe nach, wie es sich ergibt.

Sie führen eine Übersichtszystoskopie durch, registrieren alle Tumorlokalisationen und beginnen erst nach Festlegen einer Reihenfolge systematisch mit der Resektion.

Sie begutachten Blasenboden und Hinterwand, wo die Tumoren vorbeschrieben sind, und resezieren diese der Reihe nach. Tumoren an den übrigen Lokalisationen wurden vom niedergelassenen Urologen bereits ausgeschlossen.

Sie injizieren 5‑Aminolävulinsäure in die Harnblase, da Sie eben entschieden haben, die TUR‑B mit photodynamischer Diagnostik durchzuführen.

Sie besprechen gerade die präoperative Checkliste mit dem Anästhesisten vor einer transurethralen Resektion von Blasengewebe (TUR-B). Den Befund eines 4 cm großen Blasentumors an der rechten Seitenwand haben Sie selbst zystoskopisch gesichert. Unter anderem sollten Sie was besprechen?

Da eine relevante Blutung aufgrund der Größe des Tumors kaum zu vermeiden ist, sollten Blutkonserven nachgefordert werden.

Aufgrund der Tumorlokalisation besprechen Sie die Möglichkeit einer Allgemeinnarkose mit Relaxierung.

Aufgrund der Größe des Tumors sollte ein Obturatoriusblock durch die Anästhesie durchgeführt werden.

Da Sie aufgrund der Tumorlage eine mögliche intraperitoneale Perforation erwarten, bitten Sie den Anästhesisten, besonderes Augenmerk auf die Vitalparameter zu werfen.

Da aufgrund der Tumorlokalisation zur Hämostase eine möglichst ausgiebige Blasenfüllung während der Resektion notwendig ist, bitten Sie die Anästhesie, auf eine adäquate Analgesie zu achten.

Die Entwicklung der En-bloc-Resektion von Blasentumoren (ERBT) soll Probleme der konventionellen transurethralen Resektion von Blasengewebe (TUR-B) ausgleichen. Richtig ist:

Randomisierte prospektive Daten konnten zeigen, dass die Rezidivrate durch En-bloc-Resektion deutlich gesenkt werden kann.

Schlüsselkonzepte der ERBT beinhalten die verlässliche Resektion von ausreichend Detrusormuskulatur und die bessere Beurteilbarkeit von freien Schnitträndern.

Die ERBT soll laut gängigen Leitlinien nur angeboten werden, falls der Patient für eine TUR‑B nicht infrage kommt.

Die Evakuation von Blasentumoren im Ganzen nach ERBT ist bei papillären Blasentumoren problemlos gegeben.

Die ERBT ist zur Behandlung von Blasentumoren noch nicht zugelassen.

Sie beginnen eine transurethrale Resektion von Blasengewebe (TUR-B) bei einem 5 cm großen Tumor am Blasenboden. Welches Konzept ist richtig?

Sie resezieren horizontal die oberste Schicht des exopyhtischen Tumoranteils und arbeiten sich dann langsam zum Tumorgrund vor, um eine gleichmäßige Resektion zu gewährleisten.

Sie beginnen mit der Resektion am Rand des Tumors im Gesunden und resezieren immer vertikal bis zum Tumorgrund, um für eine ausreichende Hämostase zu sorgen.

Da der Tumor ohnehin schon sehr groß ist, verzichten Sie auf einen Spülstrom, um die Blase nicht zu überdehnen.

Um die Blasenwand nicht zu verletzen, aktivieren Sie die Schlinge erst im Gewebe.

Das resezierte Gewebe wird gemeinsam an die Histopathologie gesandt. Eine weitere Aufteilung ist bei einem singulären Tumor nicht notwendig.

In der Operationsbesprechung präsentieren Sie einen Patienten zur transurethralen Resektion von Blasengewebe (TUR-B) am Folgetag, der neben zystoskopisch erstmals nachgewiesenem Blasentumor von 1 cm Größe auch auffällige flächige Rötungen der Blasenschleimhaut aufweist. Was schlagen Sie vor?

Da es sich um eine Erstresektion handelt, muss keine erweiterte Tumorvisualisation stattfinden.

Durchführung der Resektion mit photodynamischer Diagnostik (PDD), da der Verdacht auf ein Carcinoma in situ gegeben ist.

Durchführung mit PDD, da die Erstdiagnose eines papillären Blasentumors gegeben ist.

Verschieben der Operation, da bei flächigen Rötungen auch bei blander Urinkultur die Möglichkeit einer Entzündung vorliegt.

Durchführung mit PDD, da durch die verbesserte Visualisierung mit Grünlicht mehr Tumor gefunden werden kann.

Sie führen eine transurethrale Resektion von Blasengewebe (TUR-B) bei einem papillären Blasentumor über dem rechten Ostium durch. Worauf achten Sie?

Das Ostium sollte nicht überreseziert werden. Im Zweifel bleiben kleine Anteile des papillären Tumors stehen und können zystoskopisch nachgesorgt werden.

Aufgrund der guten Durchblutung in diesem Bereich achten Sie auf eine ausführliche Koagulation um das Ostium.

Um eine vollständige Tumorresektion zu erreichen, resezieren Sie auch das Ostium mit, sodass kein Tumorgewebe stehen bleibt.

Nach Resektion des Ostiums legen Sie immer eine Harnleiterschiene ein, da sonst zwingend eine Harnabflussstörung entsteht.

Sonographische Nachkontrollen der Niere nach Ostiumresektion sind nicht zielführend, da bei Vermeiden von Koagulation keine Harnabflussstörungen entstehen.

Nach Ausschleusung des Patienten werden Sie angerufen, dass der Patient, bei dem Sie eben eine transurethrale Resektion von Blasengewebe (TUR-B) mit wasserklarer Spülung beendet haben, nachblutet. Wie reagieren Sie?

Sie indizieren Bluttransfusionen zur Aufrechterhaltung des Hämoglobinwerts, bis die Blutung durch Zuwarten sistiert.

Bei wiederkehrenden hellroten Fahnen im Spülstrom des Spülkatheters erkennen Sie, dass die Blutung bereits sistiert, und warten zu.

Nach Ausschluss einer Blasentamponade bzw. Evakuation derselben achten Sie auf eine ausreichende Rundspülung. Bei deutlich heller werdendem Harn warten Sie unter Blutbildkontrollen zu.

Sie indizieren die Sectio alta zur offenen Blasenexploration und Blutstillung, da die Koagulation von innen nicht gereicht hat.

Solange der Patient stabil bleibt, besteht kein Handlungsbedarf. Die Blutung ist sogar förderlich, da sie eine gute Wundheilung erahnen lässt.

Während der transurethralen Resektion von Blasengewebe (TUR-B) eines großen Tumors des Blasendachs fällt plötzlich die Blasenfüllung ab, obwohl ausreichend Spülstrom vorhanden ist. Wie reagieren Sie?

Sie beenden unmittelbar die Resektion und legen einen Katheter ein. Eine Perforation am Blasendach kann im Regelfall konservativ behandelt werden.

Sie führen die Resektion mit verminderter Blasenfüllung vorsichtig fort. Die vollständige Resektion des Blasentumors hat immer oberste Priorität.

Sie schließen den Ablauf, um für eine verbesserte Blasenfüllung zu sorgen, und fahren mit der Resektion fort.

Bei nicht eindeutig sichtbarer Perforation führen Sie eine Zystographie durch, in der sich eine intraperitoneale Perforation bestätigt. Sie indizieren die Sectio alta.

Ein Assistent hilft mit Druck auf den Bauch, um eine günstige Position zur Tumorresektion zu finden.

## References

[1] Ferlay J, Soerjomataram I, Dikshit R (2015). Cancer incidence and mortality worldwide: sources, methods and major patterns in GLOBOCAN 2012. Int J Cancer.

[2] Burger M, Catto JWF, Dalbagni G (2013). Epidemiology and risk factors of urothelial bladder cancer. Eur Urol.

[3] Babjuk M, Burger M, Compérat EM (2019). European association of urology guidelines on non-muscle-invasive bladder cancer (TaT1 and carcinoma in situ)—2019 update. Eur Urol.

[4] Deuker M, Martin T, Stolzenbach F (2021). Bladder cancer: a comparison between non-urothelial variant histology and urothelial carcinoma across all stages and treatment modalities. Clin Genitourin Cancer.

[5] Gospodarowicz M, Brierley J, Wittekind C (2017). TNM classification of malignant tumors.

[6] Eble J, Sauter G, Epstein J, Sesterhenn I (2016) Pathology and genetics of tumours of the urinary system and male genital organs, 3. Aufl. WHO classification of tumours. Lyon, France

[7] Moch H, Humphrey P, Ulbright T, Reuter V (2016) WHO classification of tumours of the urinary system and male genital organs, 4. Aufl. Lyon, France 10.1016/j.eururo.2016.02.028 26996659

[8] Brausi M, Collette L, Kurth K (2002). Variability in the recurrence rate at first follow-up cystoscopy after TUR in stage Ta T1 transitional cell carcinoma of the bladder: a combined analysis of seven EORTC studies. Eur Urol.

[9] Sylvester RJ, Rodríguez O, Hernández V (2021). European association of urology (EAU) prognostic factor risk groups for non-muscle-invasive bladder cancer (NMIBC) incorporating the WHO 2004/2016 and WHO 1973 classification systems for grade: an update from the EAU NMIBC guidelines panel. Eur Urol.

[10] Witjes JA, Bruins HM, Cathomas R (2020). European association of urology guidelines on muscle-invasive and metastatic bladder cancer: summary of the 2020 guidelines. Eur Urol.

[11] Cumberbatch MGK, Foerster B, Catto JWF (2018). Repeat transurethral resection in non-muscle-invasive bladder cancer: a systematic review. Eur Urol.

[12] Naselli A, Hurle R, Paparella S (2018). Role of restaging transurethral resection for T1 non-muscle invasive bladder cancer: a systematic review and meta-analysis. Eur Urol Focus.

[13] Retz M, Maisch P, Gschwend JE (2016). S3-Leitlinie Harnblasenkarzinom. Urologe.

[14] Baltacı S, Bozlu M, Yıldırım A (2015). Significance of the interval between first and second transurethral resection on recurrence and progression rates in patients with high-risk non-muscle-invasive bladder cancer treated with maintenance intravesical bacillus Calmette-Guérin. BJU Int.

[15] Lodde M, Palermo S, Comploj E (2005). Four years experience in bladder preserving management for muscle invasive bladder cancer. Eur Urol.

[16] Hofmann R (2018). Endoskopische Urologie – Atlas und Lehrbuch.

[17] Smith JJ, Howards S, Preminger G, Dmochowski R (2018). Hinman’s Atlas of urologic surgery.

[18] Jocham D, Miller K, Burger M, Schrader M (2020). Praxis der Urologie.

[19] Sylvester RJ, van der Meijden APM, Oosterlinck W (2006). Predicting recurrence and progression in individual patients with stage Ta T1 bladder cancer using EORTC risk tables: a combined analysis of 2596 patients from seven EORTC trials. Eur Urol.

[20] Hupe MC, Dormayer L, Ozimek T (2020). Impact of double J stenting or nephrostomy placement during transurethral resection of bladder tumour on the incidence of metachronous upper urinary tract urothelial cancer. BMC Cancer.

[21] Kramer MW, Altieri V, Hurle R (2017). Current evidence of transurethral en-bloc resection of nonmuscle invasive bladder cancer. Eur Urol Focus.

[22] Struck JP, Karl A, Schwentner C, Herrmann TRW, Kramer MW (2018). En bloc resection and vaporization techniques for the treatment of bladder cancer. Urologe A.

[23] Herrmann TRW, Wolters M, Kramer MW (2017). Transurethral en bloc resection of nonmuscle invasive bladder cancer: trend or hype. Curr Opin Urol.

[24] Neuzillet Y, Methorst C, Schneider M (2014). Assessment of diagnostic gain with hexaminolevulinate (HAL) in the setting of newly diagnosed non-muscle-invasive bladder cancer with positive results on urine cytology. Urol Oncol.

[25] Draga ROP, Grimbergen MCM, Kok ET, Jonges TN, van Swol CFP, Bosch JLHR (2010). Photodynamic diagnosis (5-aminolevulinic acid) of transitional cell carcinoma after bacillus Calmette-Guérin immunotherapy and mitomycin C intravesical therapy. Eur Urol.

[26] Ray ER, Chatterton K, Khan MS (2010). Hexylaminolaevulinate fluorescence cystoscopy in patients previously treated with intravesical bacille Calmette-Guérin. BJU Int.

[27] Rolevich AI, Zhegalik AG, Mokhort AA (2017). Results of a prospective randomized study assessing the efficacy of fluorescent cystoscopy-assisted transurethral resection and single instillation of doxorubicin in patients with non-muscle-invasive bladder cancer. World J Urol.

[28] Chou R, Selph S, Buckley DI (2017). Comparative effectiveness of fluorescent versus white light cystoscopy for initial diagnosis or surveillance of bladder cancer on clinical outcomes: systematic review and meta-analysis. J Urol.

[29] Ye Z, Hu J, Song X (2015). A comparison of NBI and WLI cystoscopy in detecting non-muscle-invasive bladder cancer: a prospective, randomized and multi-center study. Sci Rep.

[30] Drejer D, Béji S, Munk Nielsen A, Høyer S, Wrist Lam G, Jensen JB (2017). Clinical relevance of narrow-band imaging in flexible cystoscopy: the DaBlaCa-7 study. Scand J Urol.

[31] Kim SB, Yoon SG, Tae J (2018). Detection and recurrence rate of transurethral resection of bladder tumors by narrow-band imaging: prospective, randomized comparison with white light cystoscopy. Investig Clin Urol.

[32] Zheng C, Lv Y, Zhong Q, Wang R, Jiang Q (2012). Narrow band imaging diagnosis of bladder cancer: systematic review and meta-analysis. BJU Int.

[33] Naito S, Algaba F, Babjuk M (2016). The clinical research office of the endourological society (CROES) multicentre randomised trial of narrow band imaging-assisted transurethral resection of bladder tumour (TURBT) versus conventional white light imaging-assisted TURBT in primary non-muscle-invasive bladder cancer patients: trial protocol and 1-year results. Eur Urol.

[34] Kamphuis GM, de Bruin DM, Brandt MJ (2016). Comparing image perception of bladder tumors in four different Storz professional image enhancement system modalities using the íSPIES app. J Endourol.

